# Genome-Wide Association Studies Reveal Candidate Genes Associated with Pigmentation Patterns of Single Feathers of Tianfu Nonghua Ducks

**DOI:** 10.3390/ani14010085

**Published:** 2023-12-26

**Authors:** Grace Twumasi, Huazhen Wang, Yang Xi, Jingjing Qi, Liang Li, Lili Bai, Hehe Liu

**Affiliations:** 1 State Key Laboratory of Swine and Poultry Breeding Industry, College of Animal Science and Technology, Sichuan Agricultural University, Chengdu 611130, China; gracetb15@yahoo.com (G.T.); 2021302151@stu.sicau.edu.cn (H.W.); xiyang9596@163.com (Y.X.); 15512296736@163.com (J.Q.); liliang@sicau.edu.cn (L.L.); bll20ran@163.com (L.B.); 2 Farm Animal Genetic Resources Exploration and Innovation, Key Laboratory of Sichuan Province, Sichuan Agricultural University, Chengdu 611130, China

**Keywords:** duck, dorsal feather, ventral feather, color trait, pigmented region

## Abstract

**Simple Summary:**

The genetic basis underlying the pigmentation pattern of duck feathers requires extensive study. Major genes related to diverse color patterns of duck feathers remain unknown. Therefore, in this study, a genome-wide association study was used to reveal genes responsible for variations in the color pattern of single feathers of ducks. The results from this study revealed that the variation in color patterns on the left and right sides of the single feathers is associated with genes that are responsible for regulating pigment deposition and migration in the feather follicles of Tianfu Nonghua ducks. In this study, *WNT3A*, *DOCK1*, *RAB1A*, and *ALDH1A3* were identified as the candidate genes associated with different pigmentation patterns on the left and right sides of the single feather, as well as on the dorsal and ventral feathers.

**Abstract:**

In modern advanced genetics and breeding programs, the study of genes related to pigmentation in ducks is gaining much attention and popularity. Genes and DNA mutation cause variations in the plumage color traits of ducks. Therefore, discovering related genes responsible for different color traits and pigment patterns on each side of the single feathers in Chinese ducks is important for genetic studies. In this study, we collected feather images from 340 ducks and transported them into Image Pro Plus (IPP) 6.0 software to quantify the melanin content in the feathers. Thereafter, a genome-wide association study was conducted to reveal the genes responsible for variations in the feather color trait. The results from this study revealed that the pigmented region was larger in the male ducks as compared to the female ducks. In addition, the pigmented region was larger on the right side of the feather vane than on the left side in both dorsal and ventral feathers, and a positive correlation was observed among the feather color traits. Further, among the annotated genes, *WNT3A*, *DOCK1*, *RAB1A*, and *ALDH1A3* were identified to play important roles in the variation in pigmented regions of the various feathers. This study also revealed that five candidate genes, including *DPP8*, *HACD3*, *INTS14*, *SLC24A1*, and *DENND4A*, were associated with the color pigment on the dorsal feathers of the ducks. Genes such as *PRKG1*, *SETD6*, *RALYL*, and *ZNF704* reportedly play important roles in ventral feather color traits. This study revealed that genes such as *WNT3A*, DOCK1, *RAB1A*, and *ALDH1A3* were associated with different pigmentation patterns, thereby providing new insights into the genetic mechanisms of single-feather pigmentation patterns in ducks.

## 1. Introduction

Duck (*Anas platyrhynchos*) is highly valued among domestic birds reared globally for its meat, eggs, and other byproducts. China has more than 32 indigenous duck breeds [1], and each indigenous duck breed possesses a different phenotype for feather colors. Collectively, poultry feathers play important roles in several biological activities, such as flight, heat insulation, mate attraction, and skin protection [2]. Feather color appears in different pigmentation patterns in terms of size, color depth, and model. Different feather coloration in birds serves as an important trait in poultry production [3]. Feather color is a paramount characteristic that is considered in duck production and breeding [1,2].

Furthermore, feather color trait is specific to a breed of duck because it aids in the identification and differentiation of breeds, as well as influences consumer preference. This enhances the study into the mechanisms regulating feather coloration in ducks to meet consumers’ demands and preferences. Therefore, it is important to identify and understand the genetic basis of feather color pigmentation in ducks by revealing the color-related genes that can help protect the genetic resources of ducks. Previous studies have reported that genes such as endothelin B receptor-like (*EDNRB2*) is responsible for controlling plumage colors in ducks, and it has a significant impact on the variation in spot size on the body surface of ducks [1,4]. In addition, genes such as endothelin receptor type B (*EDNRB*), tyrosinase (*TYR*), and syntaxin 17 (*STX17*) were reported to be actively involved in the development of melanocyte [1,4], a key component in the synthesis of melanin, which significantly causes feather color variation. Studies have shown that genes such as *TYR*, tyrosinase-related protein 1 (*TYRP1*), melanocortin 1 receptor (*MC1R*), and agouti signaling protein (*ASIP*) play active and collective roles in the formation of plumage color and protection against ultraviolet radiation [2,5,6]. It was indicated previously that tyrosine kinase receptor (*c-kit*) and melanocyte-inducing transcription factor (*MITF*) pathways in the feather bulbs are associated with the white plumage color of ducks [7]. These show that few works have been conducted on genes and the various genetic mechanisms affecting feather pigmentation in poultry. However, there is little information available on the genetic basis of the dorsal and ventral feather color traits of ducks.

The Tianfu Nonghua duck has variations in the pigmented regions on each side of its single feather. In this duck breed, the dorsal and ventral feathers are mostly black and/or brown in the upper part and white in the lower part of the feather vane, with some feathers showing all black or white color. Considering the feather rachis as a boundary element that provides the feather bilateral symmetry, the area of the pigmented regions on the left and right sides is inconsistent. The genetic mechanism responsible for this difference is still unknown. Therefore, in this study, genome-wide association study (GWAS) was used to identify the candidate genes responsible for the different feather color traits of the Tianfu Nonghua duck breed. The findings from this study will provide valuable insights into the morphology of duck feathers, and also contribute vividly to our understanding of the genetic factors underlying duck feather coloration patterns.

## 2. Materials and Methods

### 2.1. Animals and Ethics Standards

This study was conducted to meet the specific requirements of animal welfare under the guidance of ethical regulation laid down by the Institute of Animal Genetics and Breeding, Sichuan Agricultural University, with the approval number DKY20170913. The Tianfu Nonghua ducks used in this study were raised at the Poultry Breeding Unit of Sichuan Agricultural University, Ya’an, China.

### 2.2. Experimental Animals

A total of three hundred and forty (340) Tianfu Nonghua ducks (120 days old) were obtained from the Poultry Breeding Unit of Sichuan Agricultural University, Ya’an, Sichuan Province, China. The ducks were fed and managed under the same environmental conditions. Feed and water were provided *ad libitum*. Ten feathers each were collected at the same body location from the dorsal and ventral sides of the individual ducks. Thus, a total of 6680 feathers (for both the ventral (3340) and dorsal (3340) feathers) were collected from the experimental birds and were used for the phenotypic analysis. The feathers were tightly packed into enclosed rubbers for subsequent phenotyping.

### 2.3. Phenotype Collection

**Figure 1:**
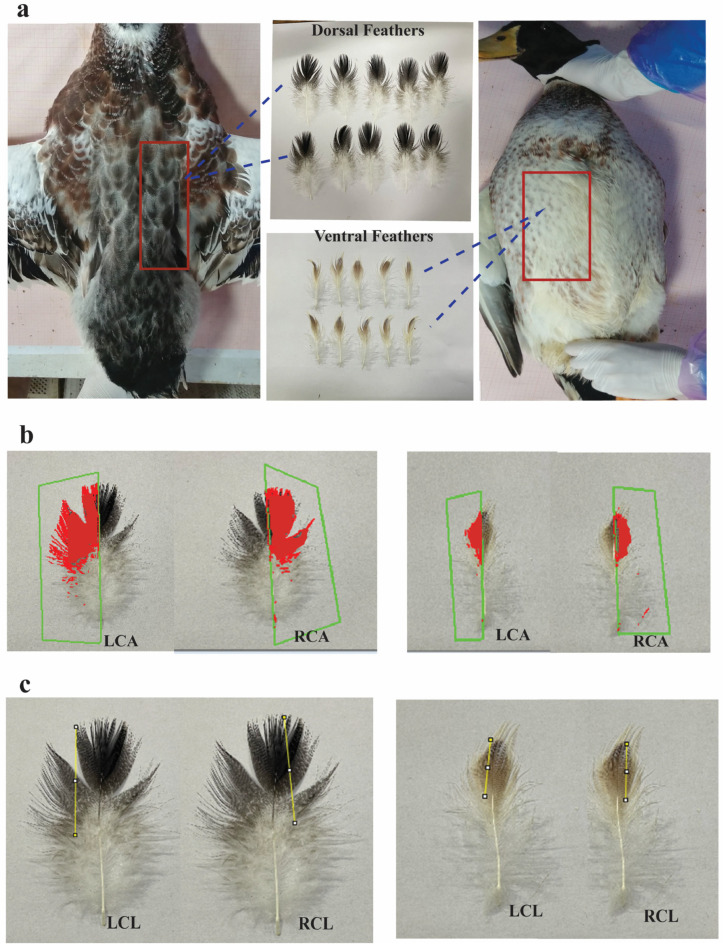
Feather types and color traits measured on dorsal and ventral feathers. (**a**) Body regions from which feathers were collected. (**b**) Measurement of left color area (LCA) and right color area (RCA) of dorsal and ventral feathers; the red shape indicates the selection of the colored area. (**c**) Measurement of left color length (LCL) and right color length (RCL) of dorsal and ventral feathers.

The feathers collected were all photographed under the same condition. Firstly, the feathers were nicely aligned side by side, and a meter rule was placed ahead of the feathers. The feathers were arranged based on similar orientations with the feather quill pointing down, as shown in Figure 1a. Using a digital camera configured for manual exposure, each set of feathers was photographed into images. Thereafter, the images were transported into the Image Pro Plus (IPP) 6.0 software (Media Cybernetics, Rockville, MD, USA) and were magnified by an identical multiplication factor. With the use of an irregular shape tool incorporated into the software, the area of interest (AOI) of each of the pigmented areas was selected, and the geometric size of each region was measured as a color area phenotype. In addition, the entire area on the left and right sides of the feather was measured. Taking the rachis or the shaft as a boundary that divides the feather into two sides, the pigmented portion on each side (left and right) of the feather of the individual ducks was measured, and then the pigmented portion of the entire feather was calculated as the sum of the pigmented portion on the left and right sides of the feather. Furthermore, the images were transported into Image Java software 1.8.0 (Image J, National Institute of Health) to measure the length of pigmented spots and the entire feather on each side of the feather vane with the use of the straight-line tool embedded in the software. All the images were treated under the same conditions. Five replicates were conducted for each measurement, and the mean values were taken as the ultimate phenotype.

Parameters measured were the left color area (LCA): the area of the color portion of the left side of the feather vane; right color area (RCA): the area of the color portion of the right side of the feather vane; total color area (TCA): the sum of the left color area and right color area; left color length (LCL): the length of the color portion of the left side of the feather; and right color length (RCL): the length of the color portion of the right side of the feather. In addition, the ratios between these parameters were considered, and the resulting parameters included the ratio of dorsal left color area to ventral left color area (DLCA: VLCA), ratio of dorsal right color area to ventral right color area (DRCA: VRCA), ratio of dorsal total color area to ventral total color area (DTCA: VTCA), and the compared values of the ratio of total color area/total feather of dorsal and ventral feathers (DTCA: TFA/VTCA: TFA).

### 2.4. Blood Sample Collection and DNA Extraction

Blood samples were collected from three hundred and forty (340) Tianfu Nonghua ducks through the primary veins and were immediately frozen at −20 °C. Then, the entire genomic DNA was isolated through a convectional phenol-chloroform protocol. The quality and quantity of the DNA were determined using Nanodrop and agarose gel electrophoresis, following the manufacturer’s instructions (Illumina, San Diego, CA, USA), [8]. The blood sample collection and DNA extraction procedures followed the guidance of the ethical regulation from the Institute of Animal Genetics and Breeding, Sichuan Agricultural University, China.

### 2.5. Genomic Resequencing, Genotyping, and SNP Calling

The process of genomic sequencing was carried out using standard procedures. Paired-end libraries were generated for each qualified sample as per the manufacturer’s protocol (Illumina, San Diego, CA, USA). The average insertion size was 500 bp, and the average read length was 150 bp. These libraries were sequenced on the Illumina^®^ Hiseq 2500 platform (Illumina, San Diego, CA, USA). The raw reads were filtered using the NGS QC (v2.3.3) Toolkit with default parameters.

Using Burrows–Wheeler alignment (BWA aln, version 0.7.12), the clean reads were mapped to the duck reference genome (ZJU 1.0) using default parameters. After mapping, single-nucleotide polymorphism (SNP) calling was performed using genome analysis toolkit (GATK) 3.5 [1,8]. The SNPs were further filtered using VCFtools (version 0.1.15) based on the following criteria: (1) SNPs with minor allele frequency > 0.05 and a major allele frequency < 0.99; (2) the maximum missing rate was <0.1; and (3) SNPs with only 2 alleles. After filtering, 4,237,004 SNPs remained and were distributed across the 29 autosomes, the Z and W sex chromosomes, and Un (unplaced scaffolds).

### 2.6. Population Structure Analysis (PCA)

The population structure of ducks was analyzed by performing PCA on all SNPs using GCTA software version 1.94.1 [9]. Three potential subgroups were identified.

### 2.7. GWAS Analysis of the Single Feather Color Trait

The SNPs that exhibited a significant association with the phenotypic traits were identified using the mixed linear model program EMMAX. The model was as follows:

y = Xα + Zβ + Wµ + e,

y is the vector of observed phenotypes including the amount of melanin pigment on each side of the single feather; Xα represents the fixed effects, including the first three principal component values (PCA eigenvectors) derived from the whole-genome SNP genotypes to correct population stratification; Zβ represents the effect of the tested SNP, with allele substitution effect β; Wµ represents the random animal effect, with variance–covariance structure based on the kinship matrix estimated using the whole-genome SNP genotypes; and e is the vector of random residual errors. Furthermore, the influence of sex was introduced as a covariate in GWAS.

QQman software in the R 3.5.1 package was used to draw Manhattan diagrams. Significance thresholds (*p*) were determined based on the Bonferroni correction, and the calculation formula was *p* = 0.05/4,237,004 SNPs (the total number of SNPs) [7]. Then, the screening criterion of the significant SNPs was calculated as −Log10*p* > 8.59. The QQ plots were generated to detect false positives resulting from population stratification. As shown in the QQ plot, the ordinate represents the observed SNP *p*-value, while the abscissa represents the theoretical *p*-value generated using a chi-squared distribution. Thus, the significant SNPs were identified by the *p*-value. Additionally, a fine-mapping analysis was conducted between the leader SNP and SNPs within the quantitative trait loci (QTL) on chr11 (Chr11: 19791413 bp; Chr11:18.79–20.79 Mbp). All the Manhattan diagrams were designed using the R programming software QQman version 3.5.1.

### 2.8. Linkage Disequilibrium (LD) Analysis

VCFtools 0.1.15 were used to extract the individual genotypes in the area of interest. Plink version 1.9 was used to conduct a linkage disequilibrium (LD) analysis between the most significant SNPs in the candidate region with a −Log10*p* value greater than 8.59. The regions were further referenced to the duck genome (ZJU I.0) to identify genes situated near the significant SNPs. Additionally, a LocusZoom graph was generated using R 3.5.1.

### 2.9. Functional Annotation of Genes

The significant SNP loci of different traits were annotated by snpEff software (version 1.9.6) with reference to the duck reference genome (ZJU1.0). The functional annotation of candidate genes was completed using the online tool KOBAS (KEGG Orthology-based annotation system), and the parameter was set to default. The false discovery rate (FDR) was corrected using the Benjamini and Hochberg method. The Gene Ontology (GO) analysis categorized the functions of genes into three distinct parts, namely biological process (BP), cellular component (CC), and molecular function (MF), along with pathways that showed *p*-values less than 0.05.

### 2.10. Statistical Analyses

Statistical analysis was conducted using the SPSS version 27.0 software to assess the differences among feather color traits. Student’s *t*-test and Pearson correlation coefficient analysis were performed to examine the relationship between the various traits. *p*-values less than 0.05 (*p* < 0.05) were considered significant.

## 3. Results

### 3.1. Phenotypic Parameters

**Figure 2:**
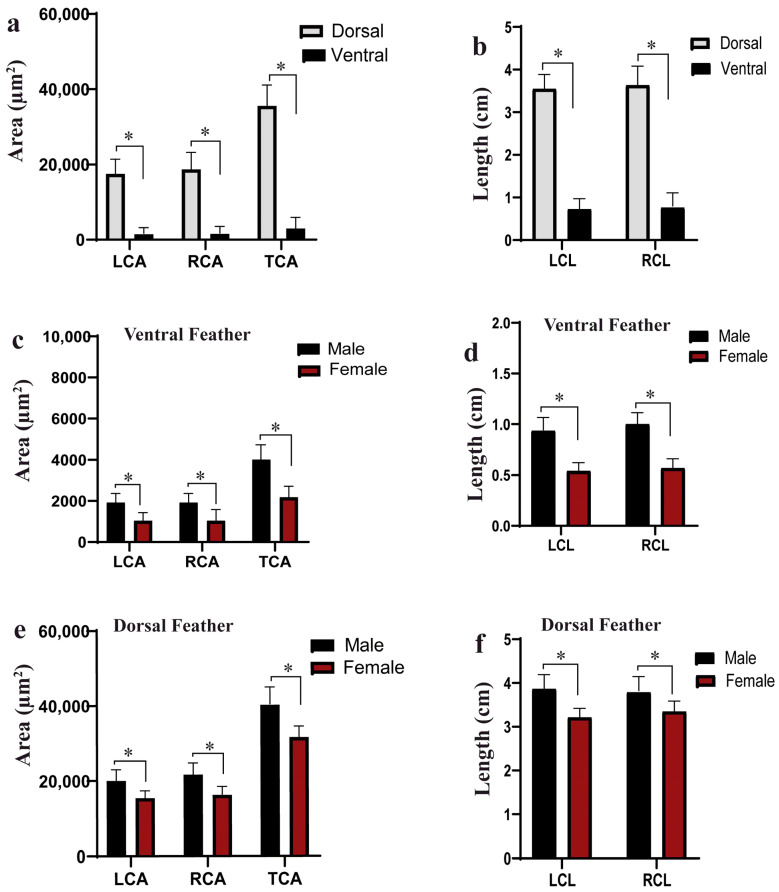
The comparison of means of the feather color phenotypes of the Tianfu Nonghua ducks. (**a**) Comparison of means of the pigmentation patterns of the dorsal and ventral feather area parameters: left color area (LCA), right color area (RCA), total color area (TCA). (**b**) Comparison of means of the pigmentation patterns of the dorsal and ventral feather length parameters: left color length (LCL), right color length (RCL). (**c**) Comparison of means of ventral feathers’ area parameters between males and females. (**d**) Comparison of means of ventral feathers’ length parameters between males and females. (**e**) Comparison of means of dorsal feathers’ area parameters between males and females. (**f**) Comparison of means of dorsal feathers’ length parameters between males and females (the asterisks mean the *p* < 0.05).

Figure 2 shows the variation in feather color traits of the Tianfu Nonghua duck. The results showed a clear distinction in the feathers for parameters such as feather color length and the depth of the color. For a detailed description of the single feather vane, Image Pro Plus version 6.0 and Image J software version 1.8.0 were employed to measure the area and length of the pigmented regions on each side of the feathers for the individual dorsal and ventral feathers of the ducks. The results further showed that the dorsal feathers appeared more pigmented than the ventral feathers (Figure 2a,b). In addition, most of the ducks had a larger portion of pigment on the right side of the feather vane compared to the left side in both the dorsal and ventral feathers (Figure 1a,b). However, in this study, 15% of the duck population showed white color for the ventral side feathers.

### 3.2. Correlation and t-Test Analysis

To ascertain the relationship between the area and length of the feather pigmentation, SPSS version 27.0 was used to calculate the correlation coefficient of the various parameters. Supplementary Tables S1–S4 showed the results of the correlations between the areas and lengths of the dorsal and ventral side feathers. We observed a positive and strong correlation among the area and length traits of the ventral feathers (Table S1). Table S2 revealed that there was a positive and moderate correlation among the area and length traits of the dorsal side feathers. This showed that the length of the feather and pigmented portion influenced the area of pigmentation in the feathers, whereas the correlation coefficients indicated the variation in the pigmented area on the left and right sides of the feather, resulted from differential pigmentation of the barb ridges during the feather growth (increase in length). Considering Tables S3 and S4, the correlation coefficient values among the dorsal and ventral side feather traits were low, indicating a weak relationship among all the traits considered. In addition, we conducted a student’s *t*-test analysis to compare the means of the area of the pigmented region between males and females. In this study, the male ducks have larger pigmented regions on the feather vane than the females for both ventral feathers (Tables S5 and S6; Figure 2c,d) and dorsal feathers (Tables S7 and S8; Figure 2e,f).

### 3.3. Population Structure

All 340 DNA samples showed good quality, with nucleic acid purity ratios of OD260/280 ≥ 1.8 and OD260/230 ≥ 2.0. These samples were used for whole-genome sequencing, and, after quality control, SNPs were retained for further analysis.

### 3.4. Significant SNPs Associated with Dorsal Feather Color Traits

**Figure 3:**
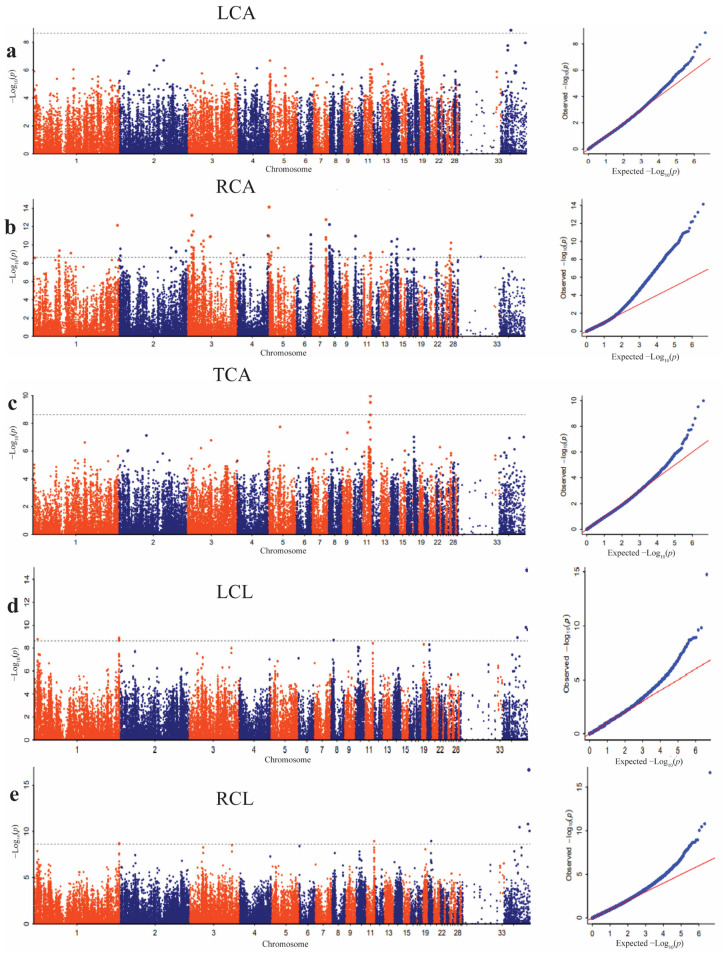
Manhattan and QQ plots for the dorsal feather traits. (**a**) Left color area (LCA). (**b**) Right color area (RCA). (**c**) Total color area (TCA). (**d**) Left color length (LCL). (**e**) Right color length (RCL). The *x*-axis shows the physical positions of each SNP along the chromosome, and the *y*-axis shows the −Log10*p* values of SNP. The gray dotted lines represent the Bonferroni threshold level (correction threshold = 8.59).

The GWAS analysis results showed that there was no high peak of SNPs for most of the pigmented areas and length traits of the dorsal feathers (Figure 3a–e). A total of 692 SNPs passed the Bonferroni significant threshold level for all the feather color traits measured on the area and length of the dorsal feathers, including the left color area, right color area, total color area, left color length, and right color length. The significant SNPs were distributed across chromosomes 1, 2, 3, 4, 5, 6, 7, 8, 10, 11, 13, 14, 16, 18, 20, 27, 32, and 34. The top significant SNPs obtained in the color traits of dorsal feathers are presented in Table 1. 

**Figure 4:**
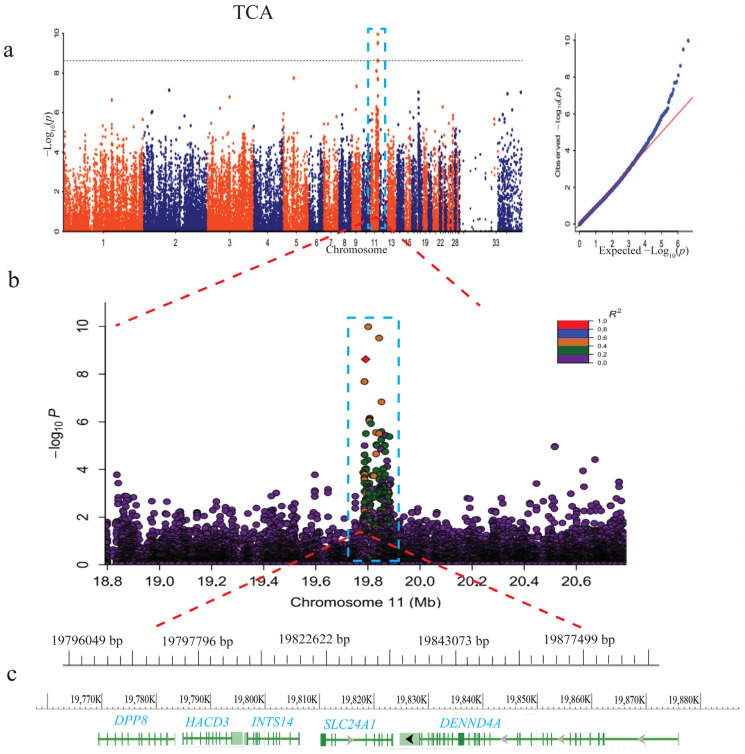
GWAS analysis of the total color area (TCA) of the dorsal side feathers. (**a**) Manhattan plot of the total color area. (**b**) LocusZoom plot for the loci ranging from 18.8 kb to 20.6 kb (the R^2^ color scale represents the different threshold of R^2^ values: purple is r^2^ > 0.0, green is r^2^ > 0.2, orange is r^2^ > 0.4, blue is r^2^ > 0.6, and red is r^2^ > 0.8). (**c**) There are five genes in the candidate region.

The Manhattan plot of the TCA showed that there was a peak of SNPs on chromosome 11 (Figure 4a). A total of three SNPs surpassed the Bonferroni significant threshold level. These SNPs were distributed in the 19791413–19843385 bp on chromosome 11. Three candidate genes, *INTS14*, *HACD3*, and *DENND4A*, were found in this region. To determine the association between the leading SNP (Chr11: 19791413 bp) and SNPs within the QTL (Chr11:18.79–20.79 Mbp), a correlation analysis was completed. The results showed that a total of 26 SNPs were highly correlated (pairwise r^2^ > 0.4; (Table S11 and Figure 4b). In this range, five candidate genes, *DPP8*, *HACD3*, *INTS14*, *SLC24A1*, and *DENND4A*, were identified (Figure 4c, Table 2).

### 3.5. Significant SNPs Associated with the Ventral Feather Color Traits

**Figure 5:**
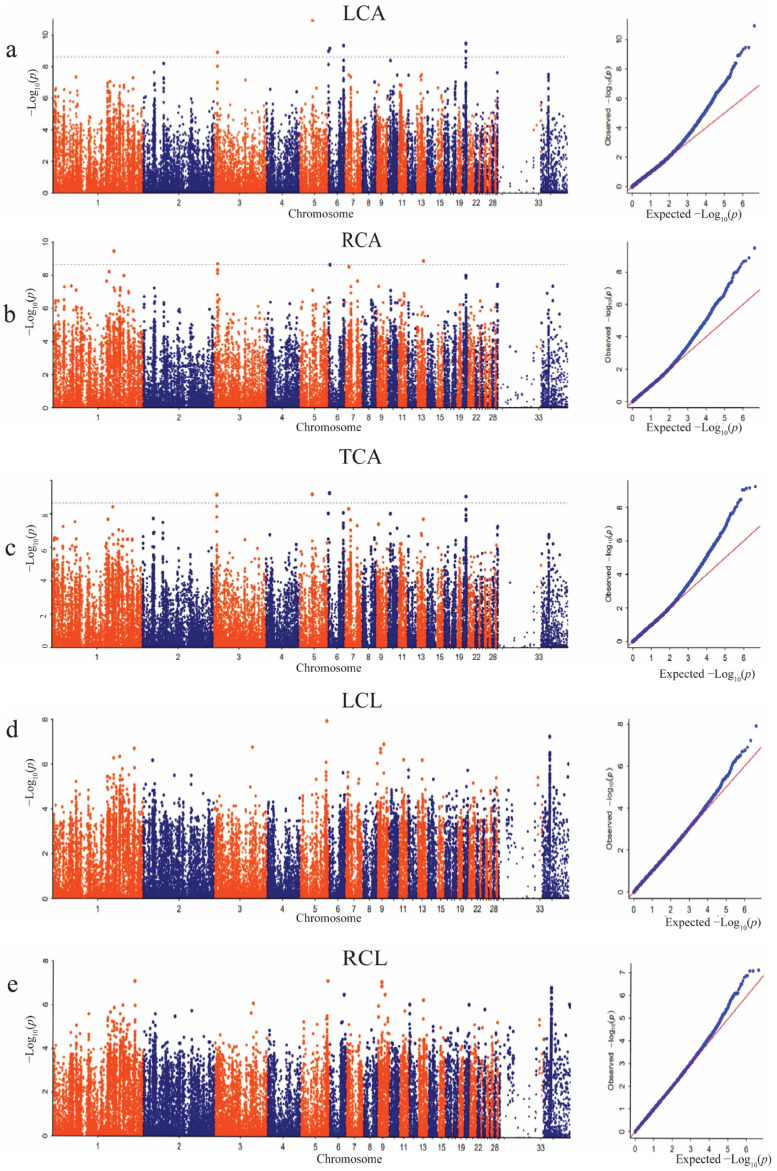
Manhattan and QQ plots for the ventral feather color traits. (**a**) Left color area (LCA). (**b**) Right color area (RCA). (**c**) Total color area (TCA). (**d**) Left color length (LCL). (**e**) Right color length (RCL). The *x*-axis depicts the physical locations of each SNP along the chromosome, while the *y*-axis depicts the −Log_10_(*p*) values of SNP. The gray dotted lines signify the Bonferroni threshold level (correction threshold = 8.59).

The results obtained from the GWAS analysis identified significant SNPs that were associated with the area and length traits measured in the ventral feathers (Figure 5). Sixty-five SNPS passed the Bonferroni significant threshold for traits measured, including left color area, right color area, and total color area. No SNP passed the Bonferroni threshold for the length traits of the ventral feathers. The significant SNPs were distributed across chromosomes 1, 3, 5, 6, 13, and 20. The top significant SNPs obtained in the color traits of ventral feathers are presented in Table 3. A total of eighteen candidate genes (*PRKG1*, *LOC113845051*, *CCDC86*, *CFAP70*, *SEC63*, *LOC101800937*, *LOC119718058*, *MACROD2*, *LOC119714415*, *PSTPIP1*, *LOC106019340*, *TSPAN3*, *KIAA0319L*, *SETD6*, *RALYL*, *ZNF704*, *SUSD6*, and *LOC101790278*) were annotated within all the significant SNPs.

### 3.6. GWAS Results of Countershading in Tianfu Nonghua Duck

The ratios of the pigmented area of the dorsal side to the ventral side feather were compared, and Student’s *t*-test analysis was performed to compare the countershading effect between the male and female ducks. The parameters measured were the ratio of dorsal left color area to ventral left color area (DLCA: VLCA), ratio of dorsal right color area to ventral right color area (DRCA: VRCA), ratio of dorsal total color area to ventral total color area (DTCA: VTCA), and the compared values of the ratio of total color area/total feather of dorsal and ventral feathers (DTCA: TFA/VTCA: TFA). The results showed that significant differences existed between the male and female ducks in all the parameters except the DRCA:VRCA. The results also showed that the effect of countershading was more prominent in female ducks than in the males (Table 4). This study also investigated the candidate genes responsible for the pigmentation pattern of the darker dorsal side and the lighter ventral side (countershading effect) feathers. Based on the GWAS results, no SNP passed the Bonferroni significant threshold level (Figure S1).

### 3.7. Functional Annotations of Genes

**Figure 6:**
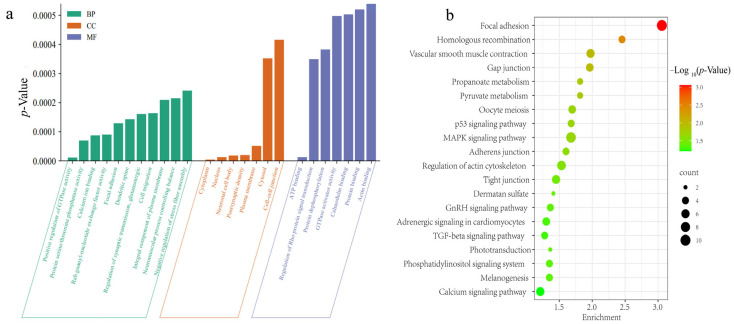
GO and KEGG enrichment analysis of the candidate genes. (**a**) GO enrichment analysis for the candidate genes. The *y*-axis indicates the *p*-values; the *x*-axis indicates the GO terms. Bars represent the *p*-value of each GO term. (**b**) KEGG enrichment analysis for the candidate genes. The *x*-axis represents the gene ratio, and the *y*-axis represents the KEGG pathways. The dot color represents the *p*-value, and the dot size represents the number of genes enriched in the reference pathway. Smaller −Log10 (*p*-values) are indicated by green, and larger values are indicated by red color.

There were 250 candidate genes identified to be associated with the significant SNPs of the dorsal feather color traits. Among these candidate genes identified, the top 25 Gene Ontology (GO) terms and the top 20 Kyoto Encyclopedia of Genes and Genomes (KEGG) pathways were shown in Figure 6 and Supplementary Tables S9 and S10, respectively. It was observed that three candidate genes, *DOCK1*, *RAB1A*, and *TMEFF2*, were significantly enriched in cell migration GO terms, whereas twenty-nine genes, *RALYL*, *ZNF704*, *TENM4*, *MEIS1*, *PURG*, *PTPRE*, *AGL*, *RGS4*, *ATF6*, *ONECUT1*, *POU2F1*, *CCNI*, *PANK4*, *ZC3H15*, *WRN*, *PKP2*, *BABAM2*, *ATF3*, *RXRG*, *PPP2CB*, *CDKL2*, *USP24*, *DOK1*, *KLF12*, *CAMTA1*, *CDK12*, *LHX9*, *DDX11*, and *MAGI2*, were significantly enriched in the nucleus GO terms. In addition, ten genes, *PCDH9*, *SCUBE3*, *LRP8*, *MASP1*, *NOTCH2*, *TBC1D9B*, *SLIT3*, *UNC13C*, *SPOCK1*, and *CAPN9*, were significantly enriched in the calcium ion binding GO terms.

The following genes, *VAV3*, *ALDH1A3*, *ASNS*, *INPP5A*, *WNT3A*, *MDH2*, *AGL*, *B4GALNT3*, *UROS*, *PANK4*, *CSGALNACT1*, *ACACA*, *GUCY2F*, and *DBT*, were enriched in at least one pathway. ASNS was enriched in three pathways, including the biosynthesis of the amino acids’ pathway, alanine, aspartate, and glutamate metabolism, and metabolic pathways. *ALDH1A*3 showed enrichment in three distinct pathways, including metabolic pathways, tyrosine metabolism pathway, and phenylalanine metabolism. Conversely, *INPP5A* was enriched in three pathways: inositol phosphate metabolism, phosphatidylinositol signaling system, and metabolic pathways. The remaining genes were enriched in either focal adhesion, melanogenesis pathway, or metabolic pathways (Table S10). In addition, eighteen candidate genes were associated with the significant SNPs of the ventral feather color traits. Among the eighteen candidate genes, four genes were enriched in the GO analysis pathways, the *PRKG1* gene was associated with the ATP binding GO terms (GO:0005524) and protein phosphorylation (GO:0006468), and three genes, including *SETD6*, *RALYL*, and *ZNF704*, were significantly enriched in the nucleus GO terms (GO:0005634).

## 4. Discussion

Plumage traits are controlled by numerous genes and molecular pathways, making them very complex to comprehend. The differences among feather color patterns are important in analyzing these molecular pathways [8]. In ducks, feather coloration is an economic and well-recognized trait. It was established in previous studies that melanin deposition, distribution, and migration in feather follicles play a significant role in feather pigmentation patterns [10]. Further studies have shown that melanin pigmentation is primarily determined by genetic factors. The study of the genetic and molecular mechanisms of plumage color has identified melanocortin 1 receptor (*MC1R*), *MITF*, and other related genes as the regulatory factors in melanocyte biology [3,10,11]. However, there are few studies on the pigmentation pattern resulting from melanin deposition and migration in single feathers of ducks. In the present study, we observed the distinct differences in coloration between the LCA and RCA, as well as between the dorsal and the ventral feathers of the Tianfu Nonghua ducks based on the quantification of melanin content using IPP. The means of the parameters measured in both the dorsal and ventral feathers were compared, and, based on this result, it was observed that the mean values of the RCA were significantly higher than in the LCA in each group of feathers. This variation in pigmentation patterns may be attributed to the genetic mechanisms that regulate feather pigment (melanin) deposition and migration. In this study, candidate genes such as *WNT3A*, *DOCK1*, *RAB1A*, and *TMEFF2* were identified to play a role in these mechanisms.

Melanocytes are responsible for producing melanin in ducks. The flow of the melanocytes is achieved through the feather follicles, which further flow into the barb ridges, where they undergo maturation, and subsequently generate pigmentation. During feather development, pigment cells situated along barb ridges transfer pigments to the neighboring keratinocytes. At the feather root level, the individual barbs are imbued with melanin that is synthesized from the melanocytes situated at the crest of the root [12]. Therefore, the assembly of many barbs with independent pigment doses on each side of the rachis causes different pigmentation patterns on the left and right sides of the feather. This genetic mechanism is stated by Xi et al., who reported that the color spots in the dorsal and ventral feathers of ducks are regulated in a dose-dependent manner by the combination of genetic loci Chr4: 10,180,939 T > C and Chr4: 10,190,671 A > T [1]. In addition, the results of the *t*-test analysis showed that the pigmented regions of the feathers in males were larger than in females. In poultry production, the difference in feather color patterns between male and female birds is believed to be influenced by genetics and sex hormones [13]. Previous research has provided evidence of the existence of estrogen receptors to play a biological role in normal human melanocytes [11]. Kim et al. discovered that estrogen can facilitate the migration of melanocytes to keratinocytes, resulting in the development of melasma [14]. In this study, the *MACROD2* (mono-ADP-ribosylhydrolase 2) gene was identified with the inclusion of sex as a covariate in the GWAS analysis. *MACROD2* is involved in the cellular pathway of adenosine, which is a precursor of adenosine diphosphate (ADP), and regulates the secretion of some sex hormones [15]. Adenosine plays a key role in pigmentation by enhancing the activity of tyrosinase, thereby increasing the melanin level in the cells. A study by Kim et al. found that low concentrations of adenosine increase the activities of melanin and tyrosinase in zebrafish [16]. Their findings support the variation in pigmentation between sexes, which may have been caused by the activity of sex hormones controlled by *MACROD2*. However, the expression level of this gene in males and females requires further study.

In this present study, a correlation analysis was performed to understand the relationship that exists between these phenotypes, and it was observed that the length of the black or brown color of the feather showed a moderate effect on the area of the pigmented region of the dorsal feathers. In addition, the length of the pigmented region of the ventral feather had a strong effect on its area measurement. This implies that, the longer the length of the pigmented regions, the larger the surface area of the pigmented spots [8,17]. The feather length is affected by the growth of the feather; therefore, the development of the feather pigment patterns is explained by the complexity of the growth (length) of the feather and the melanin deposition. This indicated that feather color in ducks is established through melanin deposition at the feather roots during development [18]. In previous studies, the concept of the diffusion–reaction model was used to explain the growth effect on the pigmentation patterns of feathers [8,12,18,19].

Further, the GWAS analysis performed on these phenotypes revealed candidate genes responsible for the color pigment of the feathers. Generally, the dorsal feathers of the duck appeared more pigmented than the ventral feathers. The GWAS results of the traits of the dorsal feathers showed candidate genes involved in the color pigments on each side and across the entire feather vane. The *WNT3A* (*Wnt* family member 3A) gene was found to be associated with the color area of the dorsal side feather. This gene belongs to the *WNT* gene family [20,21]. Through the KEGG enrichment analysis, it was revealed that *WNT3A* is enriched in the melanogenesis pathway and may be involved in the pigmentation patterns of the dorsal feathers. Previous studies have reported that *WNT3A* plays an active role in promoting melanogenesis in feather follicles by differentiating melanocyte progenitors [20,21,22]. *WNT3A* also regulates the activation of melanocyte stem cells, which transport melanin, and may initiate the Wnt/β-catenin signaling pathway, promoting the differentiation of melanocyte stem cells in the dorsal feathers. Among all the candidate genes, *DOCK1*, *RAB1A*, and *ALDH1A3* genes play crucial roles in melanogenesis and the transport of melanocytes [23].

The dedicator of cytokinesis 1 (*DOCK1*) gene is a member of the dedicator of cytokinesis protein family. It regulates the small GTPase Rac, which plays significant roles in several biological processes, including cell migration [23]. Specifically, Rac’s function is crucial in melanoblast proliferation and migration in feather follicles. Through an in vivo study using a mouse model, Li et al. found that embryonic melanoblasts with deleted Rac1 resulted in defects in migration, cell cycle progression, and cytokinesis [24]. In this present study, *DOCK1* gene was linked to the RCA, which may have improved the efficiency of pigment cell migration to increase the pigmented region on the right side compared to the left side of the feather. Previous studies have reported that Rac GTPases are essential in establishing or maintaining polarity during chemotactic migration [24,25].

Moreover, the results of the quantification of the melanin content using IPP indicated that the pigment content of the RCA and the dorsal feather was higher than that of the LCA and the ventral feather, respectively. This also explains the significant association of *RAB1A* with the RCA of dorsal feathers. *RAB1A* gene is a key regulator of protein transport in vesicles from the endoplasmic reticulum (ER) to the Golgi bodies and ultimately to the cell surface and plays a role in melanosome transport [26,27,28]. Furthermore, inclusive analysis of *RAB1A* shows that it is predominantly centralized at mature melanosomes and plays a crucial role in anterograde melanosome transport in melanocytes [26,29]. Therefore, the *RAB1A* gene is considered to be an important gene, influencing pigment deposition in dorsal feathers rather than ventral feathers [29].

The production of melanin by the melanocytes, which are situated beneath the keratinocytes, aids in the transfer of melanosomes through dendrites to the keratinocytes, where the production of melanin subsequently occurs [30,31]. Upon production, melanin is transported to the uppermost levels of the skin through the outward proliferation of keratinocytes (skin cells); *RAB1A* is considered to be involved in this process as it aids in membrane trafficking during the transfer and transport of melanin. This finding corresponds to previous studies that reported that the anterograde microtubule-dependent transport of melanosomes is regulated by *RAB1A* [30]. Also, the *RAB27A* gene (which belongs to the same family as *RAB1A*) was reported to be involved in the transfer of melanosomes to neighboring keratinocytes to enhance uniform pigmentation [32,33]. Furthermore, the KEGG analysis revealed that *ALDH1A3* (aldehyde dehydrogenase 1 family member A3) showed enrichment in metabolic pathways, the tyrosine metabolism pathway, and phenylalanine metabolism. The tyrosine metabolism plays a crucial role in the conversion of tyrosine into vital biological molecules. Specifically, tyrosine can undergo metabolism to produce hormones and also acts as a precursor for the synthesis of melanin pigment [27,34]. The *ALDH1A*3 was associated with the dorsal feather traits, which may have facilitated the production of color pigment in the dorsal feathers through the regulation of tyrosine and phenylalanine metabolism, a key component in melanogenesis [35]. These results suggest that the *DOCK1*, *RAB1A*, and *ALDH1A3* genes found in this present study may aid in the formation of the different pigmentation patterns through the regulation of melanocytes transport in feather follicles.

In addition, LocusZoom analysis of the leading SNP of TCA revealed potential genes associated with pigment deposition on feathers of the dorsal side of ducks. These candidate genes (*DPP8*, *HACD3*, *INTS14*, *SLC24A1*, and *DENND4A*) were identified to be associated with the TBA of the feather. The *DPP8* gene is a protein-coding gene and a member of the *DPP* gene family. This gene is mainly located in the cytoplasm and cytosol of an organism and is also an active component of the cytoplasm [36,37]. It is implicated in various biological processes, such as apoptosis, cell proliferation, and migration. Notably, *DPP8* is found to be expressed in cervical cancer tissues and cells of humans [27].

*HACD3* (3-Hydroxyacyl-coA dehydratase 3) gene produces a protein, and it is found in the endoplasmic reticulum and nuclear membrane. It plays a significant role in the production and breakdown of fatty acids [38,39]. In addition, *HACD3* aids insulin receptor (IR) recycling back to the plasma membrane by stimulating its association with the *RAB11A* gene. The activity of *HACD3* affects the autophosphorylation of the IR, which in turn regulates tyrosine phosphorylation [36,39], an important factor in the development of melanoma. *INTS14* is a protein-coding gene and a component of the integrator (*INT*) complex, which is important for the termination of transcription of protein-coding genes after pausing. The INT family module including the *INTS14* gene exhibits binding affinity to DNA and RNA molecules, mostly to RNA stem-loop regions. This interaction results in a strengthened association between the cleavage module and the target RNAs, thereby promoting stability in the system. [40,41]. Anemia and folic acid deficiency diseases are associated with *INTS14*. *SLC24A1* is also a protein-coding gene. It plays an important role in sodium/calcium ion transport and melanin synthesis [42]. The *SLC24A1* gene is involved in pigment-related diseases [43,44], and its mutational effect plays a role in the development of congenital stationary night blindness [44].

*DENND4A* (*DENN*-domain-containing 4A) gene produces a protein that has a *DENN* domain and plays a role in releasing guanosine diphosphates (GDP) from the small guanine nucleotide-binding proteins (G-proteins) for the formation of guanosine triphosphates (GTP). Additionally, the protein activates ras-related proteins, particularly Rab-10, which regulates intracellular vesicle trafficking [45,46]. The *DENND4A* gene also contains molecules that are vital for antiviral responses and inflammation, and may play a key role in regulating avian beak and feather disease virus (BFDV) [47]. Generally, these candidate genes were identified in the TCA of dorsal feathers, and the involvement of some of these genes in the pigmentation of feathers is unknown. However, their biological roles, such as regulation of tyrosine phosphorylation activity, sodium/calcium ion transport, termination of the transcription process, cell transport, and melanin synthesis, suggest that *DPP8*, *HACD3*, *INTS14*, *SLC24A1*, and *DENND4A* play important roles in the pigmentation of the dorsal feathers of ducks. This finding conformed to the results of previous studies, which reported that *SLC24A5* and *SLC45A2* were highly expressed in the black color tissues of mouse and dorsal feathers, thereby contributing to an increase in the tyrosinase and melanin levels in the dorsal feathers [48,49,50]. Moreover, four genes, including *PRKG1*, *SETD6*, *RALYL*, and *ZNF704*, were found to be associated with the ventral side feather color trait. *PRKG1* are important components of protein phosphorylation and signals’ transduction processes in different cell types [51,52]. The proteins phosphorylated by *PRKG1* play a key role in regulating neuronal and cardiovascular functions in addition to relaxing smooth muscle and modulating cell growth [52]. This gene is involved in many cancer diseases [52,53]. *SETD6* encodes for proteins in addition to regulating inflammatory and proliferation processes. In addition, *SETD6* controls the expression of many hormones, such as lysine and estrogen. Other previous studies revealed that *RALYL* and *ZNF704* are involved in melanocyte cell differentiation and proliferation [54]. Therefore, these genes may play a key role in the pigmentation pattern of ventral feathers; however, their genetic mechanisms in feather pigmentation require further study.

Furthermore, dorsoventral (DV) countershading observed in ducks is a pigmentary adaptation that is remarkably conserved and plays a crucial role in camouflage, predation, and protection against solar radiation. The ducks used in this study showed darker dorsal feathers and lighter ventral feather color. Based on the comparison analysis, the effect of countershading was more prominent in the female than in the male ducks. The ratio of the dorsal to ventral feather pigmentation was larger in females than in males due to the lighter ventral feather colors of the female ducks. This result supports the well-established fact that male birds are more colorful than female birds [12,55]. In this study, no candidate gene was identified to be associated with the countershading effect in the Tianfu Nonghua ducks; however, previous studies showed that the expression between melanocortin receptors (*MC1R*) and agouti signaling protein *ASIP* genes controls countershading in vertebrates [2,56,57].

## 5. Conclusions

Taken together, the results obtained in this study indicated that the right side of the duck’s feather vane showed a larger pigment portion as compared to the left side of both the dorsal and ventral side feathers. In addition, we found that *DPP8*, *HACD3*, *INTS14*, *SLC24A1*, and *DENND4A* were the main candidate genes associated with the total color area of the dorsal side feather, and *WNT3A*, *DOCK1*, *RAB1A*, and *ALDH1A3* genes are responsible for variation in pigmented regions between the left and right color area as well as between the dorsal and ventral side feathers. It was also revealed in this study that *PRKG1*, *SETD6*, *RALYL*, and *ZNF704* were significantly associated with the ventral feather color traits. Therefore, these results provided valuable insights into the morphology of duck feathers, as well as contributed to our understanding of the genetic factors underlying the coloration patterns of duck feathers.

## Figures and Tables

**Table 1:** The top significant single-nucleotide polymorphisms (SNPs) identified in dorsal feather color traits.

Trait	Chr	Position	−Log_10_(*p*)	REF	ALT	Gene
RCA	Chr3	11106947	16.928888	C	A	*CEP68*
	Chr1	203570330	12.13432	C	A	*TENM4*
	chr3	39330988	10.44989	T	C	*LOC101794856*
	chr3	39517020	9.794113	A	G	*LOC101795266*
	chr3	54977399	10.88282	C	T	*ARID1B*
	chr3	56297389	10.91372	C	T	*AIG1*
	chr4	16056736	8.885082	T	C	*PURG*
TCA	chr11	19801194	9.981866	G	A	*INTS14*
	chr11	19843385	9.511955	C	T	*HACD3*
	chr11	19791413	8.619472	G	A	*DENND4A*
LCL	chr1	204456821	8.877868	G	A	*GUCY2F*
	chr1	204456829	8.858735	A	G	*GUCY2F*
	chr1	204456818	8.714282	G	A	*GUCY2F*
	chr8	7523520	8.704662	T	A	*KCNT2*
RCL	chr20	7466942	8.96404	T	C	*MYL10*
	chr11	19969233	8.951823	T	C	*MEGF11*
	chr1	204456818	8.719696	G	A	*GUCY2F*

Chr = chromosome, *p* = probability of main effect, REF = the base of the reference genome, ALT = the mutated base, RCA = right color area, TCA = total color area, LCL = left color length, RCL = right color length.

**Table 2:** Genes identified in the candidate region of chromosomes Chr11 of the total color area for the dorsal side feather.

Chr	Position	*p*-Value	REF	ALT	Gene
chr11	19796049	0.000434	G	A	*DPP8*
chr11	19797796	0.023138	T	A	*HACD3*
chr11	19822622	0.000182	G	T	*INTS14*
chr11	19843073	0.000000	G	A	*SLC24A1*
chr11	19877499	0.001007	G	A	*DENND4A*

Chr = chromosome, *p* = probability of main effect, REF = the base of the reference genome, ALT = the mutated base.

**Table 3:** The top significant SNPs identified in the ventral feather color traits.

Trait	Chr	Pos	−Log_10_(*p*)	REF	ALT	Gene
LCA	chr5	30633861	10.94515	G	A	*CCDC86*
	chr11	9151176	10.9137	G	A	*LOC119718058*
	chr3	7298398	10.51952	G	A	*MACROD2*
	chr6	817341	8.982077	T	G	*PRKG1*
RCA	chr1	140523618	9.463266	A	G	*-*
	chr13	15682406	8.862459	T	C	*LOC113845051*
	chr3	7616580	8.693525	T	C	*MACROD2*
	chr6	3619856	8.638454	C	G	*LOC101796401*
TCA	chr6	3619856	9.240001	C	G	*LOC101796401*
	chr5	30633861	9.162349	G	A	*CCDC86*
	chr3	7616580	9.131946	T	C	*MACROD2*

Chr = chromosome, *p* = probability of main effect, REF = the base of the reference genome, ALT = the mutated base, LCA = left color area, RCA = right color area, TCA = total color area.

**Table 4:** *t*-test results of the countershading effect between the male and female ducks.

Parameters	Male, n = 156		Female, n = 171		t	95% CI		*p*
	M	SD	M	SD		Lower	Upper	
DLCA: VLCA	16.89	52.34	40.49	117.30	−2.04	−46.3263	−0.87246	0.04
DRCA: VRCA	19.21	93.69	49.58	181.71	−1.66	−66.4726	5.732883	0.09
DTCA: VTCA	12.93	19.10	48.66	181.08	−2.14	−68.5645	−2.90038	0.03
DTCA:TFA/VTCA: TFA	7.39	16.45	37.22	148.99	−2.17	−56.86	−2.81	0.03

M = mean; SD = standard deviation; t = *t*-test value; CI = confidence interval; *p* = probability value; DLCA: VLCA = ratio of dorsal left color area to ventral left color area; DRBA: VRCA = ratio of dorsal right color area to ventral right color area; DTCA: VTCA = ratio of dorsal total color area to ventral total color area; DTCA: TFA/VTCA: TFA = compared values of the ratio of total color area/total feathers of dorsal and ventral feathers.

## Data Availability

The datasets used and analyzed during the current study are available from the corresponding author on request. The data are not publicly available due to ethical restrictions. The datasets supporting this article are included in the main manuscript, while Supplemental Materials such as tables and figures are attached to the manuscript.

## Supplementary Materials

The following supporting information can be downloaded at https://www.mdpi.com/article/10.3390/ani14010085/s1, Figure S1: Manhattan and QQ plots for countershading parameters. (a) DLCA: VLCA. (b) DRCA: VRCA. (c) DTCA: VTCA. (d) DTCA: TFA/VTCA: TFA. The *x*-axis shows the physical positions of each SNP along the chromosome, and the *y*-axis shows the −Log_10_(*p*) values of SNP. The gray dotted lines represent the Bonferroni threshold level (correction threshold = 8.59).; Table S1: Correlation results for ventral side feathers’ traits; Table S2: Correlation results for dorsal side feathers’ traits; Table S3: Correlation results for dorsal and ventral side feathers’ area traits; Table S4: Correlation results for dorsal and ventral side feathers’ length traits; Table S5: *t*-test results of ventral feather area traits; Table S6: *t*-test results of ventral feather length traits; Table S7: *t*-test results of dorsal feather area traits; Table S8: “*t*-test”results of dorsal feather length traits; Table S9: Results of GO enrichment analysis for identified candidate genes; Table S10: Results of KEGG enrichment pathway for identified associated genes; Table S11: SNPs with a pairwise r^2^ > 0.4 with the leader SNP at chr11: 19791413 bp.

## Abbreviations

Chr	Chromosome
LCL	Left color length
RCL	Right color area
TCA	Total color area
LCL	Left color length
RCL	Right color length
BP	Biological process
CC	Cellular component
MF	Molecular function
IPP	Image Pro Plus
AOI	Area of interest
QTL	Quantitative trait loci
LD	Linkage disequilibrium
KEGG	Kyoto Encyclopedia of Genes and Genomes
GO	Gene Ontology
DNA	Deoxyribonucleic acid
SNP	Single-nucleotide polymorphism
GWAS	Genome-wide association studies
VCF	Variant call format
PCA	Principal component analysis
QQ	Quantile–quantile
ADP	Adenosine diphosphate

## References

[1] Xi Y., Xu Q., Huang Q., Ma S., Wang Y., Han C., Zhang R., Wang J., Liu H., Li L. (2021). Genome-wide association analysis reveals that EDNRB2 causes a dose-dependent loss of pigmentation in ducks. BMC Genom..

[2] Xi Y., Liu H., Li L., Xu Q., Liu Y., Wang L., Ma S., Wang J., Bai L., Zhang R. (2020). Transcriptome Reveals Multi Pigmentation Genes Affecting Dorsoventral Pattern in Avian Body. Front. Cell Dev. Biol..

[3] Zhou Z., Li M., Cheng H., Fan W., Yuan Z., Gao Q., Xu Y., Guo Z., Zhang Y., Hu J. (2018). An intercross population study reveals genes associated with body size and plumage color in ducks. Nat. Commun..

[4] Sun Y., Wu Q., Lin R., Chen H., Zhang M., Jiang B., Wang Y., Xue P., Gan Q., Shen Y. (2023). Genome-wide association study for the primary feather color trait in a native Chinese duck. Front. Genet..

[5] Kerje S., Lind J., Schütz K., Jensen P., Andersson L. (2003). Melanocortin 1-receptor (MC1R) mutations are associated with plumage colour in chicken. Anim. Genet..

[6] Hiragaki T., Inoue-Murayama M., Miwa M., Fujiwara A., Mizutani M., Minvielle F., Ito S. (2008). Recessive black Is Allelic to the yellow Plumage Locus in Japanese Quail and Associated With a Frameshift Deletion in the ASIP Gene. Genetics.

[7] Li S., Wang C., Yu W., Zhao S., Gong Y. (2012). Identification of Genes Related to White and Black Plumage Formation by RNA-Seq from White and Black Feather Bulbs in Ducks. PLoS ONE.

[8] Ma S., Li P., Liu H., Xi Y., Xu Q., Qi J., Wang J., Li L., Wang J., Hu J. (2023). Genome-wide association analysis of the primary feather growth traits of duck: Identification of potential Loci for growth regulation. Poult. Sci..

[9] Yang J., Lee S.H., Goddard M.E., Visscher P.M. (2011). GCTA: A tool for genome-wide complex trait analysis. Am. J. Hum. Genet..

[10] Yu W., Wang C., Xin Q., Li S., Feng Y., Peng X., Gong Y. (2013). Non-synonymous SNPs in MC1R gene are associated with the extended black variant in domestic ducks (Anas platyrhynchos). Anim. Genet..

[11] Sultana H., Seo D.W., Park H.B., Choi N.R., Hoque R., Bhuiyan S.A., Heo K.N., Lee S.H., Lee J.H. (2017). Identification of MC1R SNPs and their Association with Plumage Colors in Asian Duck. J. Poult. Sci..

[12] Yoshioka S., Akiyama T. (2021). Mechanisms of Feather Structural Coloration and Pattern Formation in Birds. Pigments, Pigment Cells and Pigment Patterns.

[13] Saino N., Romano M., Rubolini D., Teplitsky C., Ambrosini R., Caprioli M., Canova L., Wakamatsu K. (2013). Sexual dimorphism in melanin pigmentation, feather coloration and its heritability in the barn swallow (Hirundo rustica). PLoS ONE.

[14] Kim N.H., Cheong K.A., Lee T.R., Lee A.Y. (2012). PDZK1 upregulation in estrogen-related hyperpigmentation in melasma. J. Investig. Dermatol..

[15] Crawford K., Oliver P.L., Agnew T., Hunn B.H.M., Ahel I. (2021). Behavioural Characterisation of Macrod1 and Macrod2 Knockout Mice. Cells.

[16] Kim M.Y., Lee H.E., Im M., Lee Y., Kim C.D., Lee J.H., Seo Y.J. (2014). Effect of adenosine on melanogenesis in b16 cells and zebrafish. Ann. Dermatol..

[17] Chen C.F., Foley J., Tang P.C., Chuong C. (2015). Development, Regeneration, and Evolution of Feathers. Annu. Rev. Anim. Biosci..

[18] Jenni L., Ganz K., Milanesi P., Winkler R. (2020). Determinants and constraints of feather growth. PLoS ONE.

[19] Prum R.O., Williamson S. (2002). Reaction-diffusion models of within-feather pigmentation patterning. R. Soc. Proc. B.

[20] Guo H., Xing Y., Liu Y., Luo Y., Deng F., Yang T., Yang K., Li Y. (2016). Wnt/β-catenin signaling pathway activates melanocyte stem cells in vitro and in vivo. J. Dermatol. Sci..

[21] Guo H., Yang K., Deng F., Ye J., Xing Y., Li Y., Lian X., Yang T. (2012). Wnt3a promotes melanin synthesis of mouse hair follicle melanocytes. Biochem. Biophys. Res. Commun..

[22] Dongkyun K., Jinsoo S., Jin E.J. (2010). Wnt-3 and Wnt-3a play different region-secific roles in neural crest development in avians. Cell Biol. Int..

[23] Chiang S.K., Chang W.C., Chen S.E., Chang L.C. (2019). DOCK1 Regulates Growth and Motility through the RRP1B-Claudin-1 Pathway in Claudin-Low Breast Cancer Cells. Cancers.

[24] Li A., Ma Y., Yu X., Mort R.L., Lindsay C.R., Stevenson D., Strathdee D., Insall R.H., Chernoff J., Snapper S.B. (2011). Rac1 drives melanoblast organization during mouse development by orchestrating pseudopod- driven motility and cell-cycle progression. Dev. Cell.

[25] Steffen A., Ladwein M., Dimchev G.A., Hein A., Schwenkmezger L., Arens S., Ladwein K.I., Margit Holleboom J., Schur F., Victor Small J. (2013). Rac function is crucial for cell migration but is not required for spreading and focal adhesion formation. J. Cell Sci..

[26] Li Z., Li Y., Jia Y., Ding B., Yu J. (2020). Rab1A knockdown represses proliferation and promotes apoptosis in gastric cancer cells by inhibition of mTOR/p70S6K pathway. Arch. Biochem. Biophys..

[27] Chen Y., Liu F., Wu K., Wu W., Wu H., Zhang W. (2018). Targeting dipeptidyl peptidase 8 genes inhibits proliferation, migration and invasion by inhibition of cyclin D1 and MMP2MMP9 signal pathway in cervical cancer. J. Gene Med..

[28] Cheng Z., Shao X., Xu M., Wang J., Kuai X., Zhang L., Wu J., Zhou C., Mao J. (2019). Rab1A promotes proliferation and migration abilities via regulation of the HER2/AKT-independent mTOR/S6K1 pathway in colorectal cancer. Oncol. Rep..

[29] Ishida M., Ohbayashi N., Fukuda M. (2015). Rab1A regulates anterograde melanosome transport by recruiting kinesin-1 to melanosomes through interaction with SKIP. Sci. Rep..

[30] Zhang X., Zhu T., Wang L., Lv X., Yang W., Qu C., Li H., Wang H., Ning Z., Qu L. (2023). Genome-Wide Association Study Reveals the Genetic Basis of Duck Plumage Colors. Genes.

[31] Videira I.F., Moura D.F., Magina S. (2013). Mechanisms regulating melanogenesis. An. Bras. Dermatol..

[32] Hume A.N., Ushakov D.S., Tarafder A.K., Ferenczi M.A., Seabra M.C. (2007). Rab27a and MyoVa are the primary Mlph interactors regulating melanosome transport in melanocytes. J. Cell Sci..

[33] Chen D., Guo J., Miki T., Tachibana M., Gahl W.A. (1997). Molecular cloning and characterization of rab27a and rab27b, novel human rab proteins shared by melanocytes and platelets. Biochem. Mol. Med..

[34] Rzepka Z., Buszman E., Beberok A., Wrześniok D. (2016). From tyrosine to melanin: Signaling pathways and factors regulating melanogenesis. Postepy Hig. Med. Dosw. (Online).

[35] Jimbow K., Gomez P.F., Toyofuku K., Chang D., Miura S., Tsujiya H., Park J.S. (1997). Biological role of tyrosinase related protein and its biosynthesis and transport from TGN to stage I melanosome, late endosome, through gene transfection study. Pigment. Cell Res..

[36] Ajami K., Abbott C.A., Obradovic M., Gysbers V., K?Hne T., Mccaughan G.W., Gorrell M.D. (2003). Structural requirements for catalysis, expression, and dimerization in the CD26/DPIV gene family. Biochemistry.

[37] Okondo M.C., Johnson D.C., Sridharan R., Go E.B., Chui A.J., Wang M.S., Poplawski S.E., Wu W., Liu Y., Lai J.H. (2017). DPP8 and DPP9 inhibition induces pro-caspase-1-dependent monocyte and macrophage pyroptosis. Nat. Chem. Biol..

[38] Cieslak M., Reissmann M., Hofreiter M., Ludwig A. (2011). Colours of domestication. Biol. Rev. Camb. Philos Soc..

[39] Boutchueng-Djidjou M., Belleau P., Bilodeau N., Fortier S., Bourassa S., Droit A., Elowe S., Faure R.L. (2018). A type 2 diabetes disease module with a high collective influence for Cdk2 and PTPLAD1 is localized in endosomes. PLoS ONE.

[40] Sabath K., Stäubli M.L., Marti S., Leitner A., Moes M., Jonas S. (2020). INTS10-INTS13-INTS14 form a functional module of Integrator that binds nucleic acids and the cleavage module. Nat. Commun..

[41] Pfleiderer M.M., Galej W.P. (2021). Structure of the catalytic core of the Integrator complex. Mol. Cell.

[42] Gaudel C., Soysouvanh F., Leclerc J., Bille K., Husser C., Montcriol F., Bertolotto C., Ballotti R. (2020). Regulation of Melanogenesis by the Amino Acid Transporter SLC7A5. J. Investig. Dermatol..

[43] Neuillé M., Malaichamy S., Vadalà M., Michiels C., Condroyer C., Sachidanandam R., Srilekha S., Arokiasamy T., Letexier M., Démontant V. (2016). Next-generation sequencing confirms the implication of SLC24A1 in autosomal-recessive congenital stationary night blindness. Clin. Genet..

[44] Carrigan M., Duignan E., Malone C.P., Stephenson K., Saad T., McDermott C., Green A., Keegan D., Humphries P., Kenna P.F. (2016). Panel-Based Population Next-Generation Sequencing for Inherited Retinal Degenerations. Sci. Rep..

[45] Neelagandan N., Gonnella G., Dang S., Janiesch P.C., Miller K.K., Küchler K., Marques R.F., Indenbirken D., Alawi M., Grundhoff A. (2019). TDP-43 enhances translation of specific mRNAs linked to neurodegenerative disease. Nucleic Acids Res..

[46] Guo Q., Jiang Y., Wang Z., Bi Y., Chen G., Bai H., Chang G. (2022). Genome-Wide Analysis Identifies Candidate Genes Encoding Feather Color in Ducks. Genes.

[47] Desingu P.A., Nagarajan K. (2022). Detection of beak and feather disease virus in India and its implications. Transbound Emerg Dis.

[48] Gunnarsson U., Hellström A.R., Tixier-Boichard M., Minvielle F., Bed’hom B., Ito S., Jensen P., Rattink A., Vereijken A., Andersson L. (2007). Mutations in SLC45A2 cause plumage color variation in chicken and Japanese quail. Genetics.

[49] Li R., Wang X., Wang Y., Liu D., Zhang Y., Liu Y., Niu X., Han R., Li H., Jiang R. (2023). Research Note: Combined analysis of BSA-seq based mapping and RNA-seq reveals candidate genes associated with sub-Columbian plumage in H line chickens. Poult. Sci..

[50] Li R., Wang Y., Liu Y., Li D., Tian Y., Liu X., Kang X., Li Z. (2023). Effects of SLC45A2 and GPNMB on Melanin Deposition Based on Transcriptome Sequencing in Chicken Feather Follicles. Animals.

[51] Yang L., Mo C., Shen W., Du X., Akbar Bhuiyan A., Li L., Li N., Gong Y., Li S. (2019). The recessive C locus in the MITF gene plays a key regulatory role in the plumage colour pattern of duck (Anas platyrhynchos). Br. Poult. Sci..

[52] Lan T., Li Y., Wang Y., Wang Z.C., Mu C.Y., Tao A.B., Gong J.L., Zhou Y., Xu H., Li S.B. (2023). Increased endogenous PKG I activity attenuates EGF-induced proliferation and migration of epithelial ovarian cancer via the MAPK/ERK pathway. Cell Death Dis..

[53] Wang X., Nudds R.L., Palmer C., Dyke G.J. (2012). Size scaling and stiffness of avian primary feathers: Implications for the flight of Mesozoic birds. J. Evol. Biol..

[54] Luo J., Li H., Xiu J., Zeng J., Feng Z., Zhao H., Li Y., Wei W. (2023). Elevated ZNF704 expression is associated with poor prognosis of uveal melanoma and promotes cancer cell growth by regulating AKT/mTOR signaling. Biomark. Res..

[55] Hashimoto H., Goda M., Futahashi R., Kelsh R., Akiyama T. (2021). Pigments, Pigment Cells and Pigment Patterns.

[56] Cal L., Suarez-Bregua P., Comesaña P., Owen J., Braasch I., Kelsh R., Cerdá-Reverter J.M., Rotllant J. (2019). Countershading in zebrafish results from an Asip1 controlled dorsoventral gradient of pigment cell differentiation. Sci. Rep..

[57] Cal L., Suarez-Bregua P., Braasch I., Irion U., Kelsh R., Cerdá-Reverter J.M., Rotllant J. (2019). Loss-of-function mutations in the melanocortin 1 receptor cause disruption of dorso-ventral countershading in teleost fish. Pigment. Cell Melanoma Res..

