# Supplementary material for: Genome-Wide Association Studies Reveal Candidate Genes Associated with Pigmentation Patterns of Single Feathers of Tianfu Nonghua Ducks
Source: Animals (Basel). 2023 Dec 26;14(1):85. doi: 10.3390/ani14010085 (PMC10778472; doi:10.3390/ani14010085)
Supplement: Supplementary file 1 [file animals-14-00085-s001.zip › animals-2711418-supplementary.pdf]

Supplementary Tables and Figures

Table S1: Correlation results for ventral side feathers traits.

Traits	LCA	RCA	TCA	LCL	RCL
LCA	1				
RCA	0.92**	1			
TCA	0.98**	0.98**	1		
LCL	0.78**	0.77**	0.79**	1	
RCL	0.77**	0.77**	0.79**	0.98**	1

** Correlation is significant at 0.01 significant level, LCA =Left color area, RCA= Right color area, TCA = Total color area, LCL= left color length, RCL=Right color length.

Table S2: Correlation results for dorsal side feather traits.

Traits	LCA	RCA	TCA	LCL	RCL
LCA	1				
RCA	0.69**	1			
TCA	0.89**	0.86**	1		
LCL	0.44**	0.37**	0.41**	1	
RCL	0.48**	0.40**	0.45**	0.96**	1

** Correlation is significant at 0.01 significant level, LCA =Left color area, RCA= Right color area, TCA = Total color area, LCL= left color length, RCL=Right color length

Table S3: Correlation results for dorsal and ventral side feathers area traits.

Traits	DLCA	DRCA	DTCA	VLCA	VRCA	VTCA
DLCA	1					
DRCA	0.69**	1				
DTCA	0.89**	0.86**	1			
VLCA	-0.04	0.04	0.05	1		
VRCA	0.02	0.04	0.02	0.92**	1	
VTCA	0.04	0.04	0.03	0.98**	0.98**	1

** Correlation is significant at 0.01 significant level, DLCA = Dorsal left color area, DRCA= Dorsal Right color area, DTCA = Dorsal Total color area, VLCA= Ventral left color area, VRCA= Ventral right color area, VTCA= Ventral total color area.

Table S4: Correlation results for dorsal and ventral side feathers length traits.

Traits	VLCL	VRCL	DLCL	DRCL
VLCL	1			
VRCL	0.98**	1		
DLCL	0.05**	0.03	1	
DRCL	-0.03	0.02	0.96**	1

** Correlation is significant at 0.01 significant level, VLCL= Ventral left color length, VRCL= Ventral right color length, DLCL= Dorsal left color length, DRCL= Dorsal Right color length.

Table S5: T-test results of Ventral feather area traits

	Male, n=156	Female, n=171	**95% CI**	**P**

**Parameters**	M (μm²)	SD	M (μm²)	SD	**t**	**Lower**	**Upper**	
LCA	1924.1	1773.02	1041.51	2058.65	4.15	464.38	1300.92	0.00
LTA	13297.8	3622.56	11856.46	3758.10	3.50	639.24	2242.89	0.00
RCA	2084.203	1949.01	1137.02	2203.59	4.12	494.51	1399.86	0.00
RTA	14119.03	4084.77	12631.1	4397.84	3.17	565.16	2410.56	0.002
TCA	4008.34	3623.36	2178.49	4189.99	4.22	977.09	2682.58	0.00
TFA	27441.76	7250.93	24488.79	7570.50	3.60	1339.67	4566.28	0.00
Ratios								
LCA: LTA	0.15	0.14	0.09	0.17	3.33	0.023	0.092	0.001
RCA: RTA	0.15	0.14	0.09	0.169	3.41	0.025	0.092	0.001
TCA:TFA	0.15	0.14	0.09	0.17	3.45	0.025	0.092	0.001

M =Mean; SD =Standard Deviation; t = t-test value; CI = Confidence Interval**;** P =Probability value; LCA =Left color area; LTA =Left total area; RCA= Right color area; RTA = Right total area; TCA = Total color area; TFA = Total feather area; LCA:LTA= ratio of left color area to left total area; RCA:RTA= ratio of right color area to right total area; TCA:TFA = ratio of total color area to total feather.

Table S6: T-test results of Ventral feather length traits

**Parameters**	Male, n=156		Female, n=171		**t**	**95% CI**		**P**
	M (cm)	SD	M (cm)	SD		**Lower**	**Upper**	
LCL	0.94	0.69	0.54	0.76	5.03	0.25	0.56	0.00
RCL	1.00	0.73	0.57	0.80	5.12	0.27	0.60	0.00
FL	3.84	0.49	3.53	0.55	5.42	0.20	0.42	0.00
FRL	0.32	0.21	0.29	0.22	1.43	-0.01	0.08	0.155
TFL	4.17	0.45	3.82	0.56	6.13	0.23	0.46	0.00
**Ratios**								
LCL :FL	0.25	0.17	0.16	0.21	3.99	0.04	0.13	0.00
RCL :FL	0.26	0.19	0.18	0.25	3.49	0.04	0.13	0.001
LCL : TFL	0.23	0.16	0.15	0.20	3.78	0.04	0.12	0.00
RCL: TFL	0.24	0.17	0.16	0.24	3.26	0.03	0.12	0.001
FRL:TFL	0.08	0.05	0.07	0.05	0.65	-0.007	0.014	0.519

FL: TFL	0.92	0.05	0.93	0.05	-0.65	-0.014	0.007	0.519

M =Mean; SD =Standard Deviation; t = t-test value; CI = Confidence Interval; P =Probability value; LCL= left color length; RCL=Right color length; FL= Feather length; FRL= Feather root length; TFL= Total feather length; LCL:FL= ratio of left color length to feather length; RCL:FL= ratio of right color length to feather length; LCL:TFL = ratio of left color length to total feather length; RCL:TFL= ratio of right color length to total feather length; FRL:TFL = ratio of feather root length to total feather length; FL:TFL= ratio of feather length to total feather length.

Table S7: T-test results of dorsal feather area traits.

	Male, n=142		Female, n=178			**95% CI**		**P**
**Parameters**	M (μm²)	SD	M (μm²)	SD	**t**	**Lower**	**Upper**	
LCA	20011.035	8038.03	15416.27	4147.17	6.61	3226.45	5963.67	0.00
LTA	34694.83	12197.80	28518.18	7636.606	5.53	3980.36	8372.92	0.00
RCA	21696.52	11241.68	16260.29	5431.64	5.57	3551.89	7320.58	0.00
RTA	35837.52	12891.26	30380.97	9722.05	4.32	2968.73	7944.36	0.00
TCA	40287.50	15716.40	31619.44	9218.38	6.15	5895.84	11440.26	0.00
TFA	70532.34	24309.99	58899.15	16253.54	5.11	7155.76	16110.61	0.00
**Ratios**								
LCA : LTA	0.58	0.13	0.56	0.12	2.13	0.002	0.060	0.034
RCA: RTA	0.62	0.20	0.57	0.16	2.45	0.009	0.091	0.015
TCA:TFA	0.60	0.15	0.56	0.12	2.85	0.013	0.074	0.005

M =Mean; SD =Standard Deviation; t = t-test value; CI = Confidence Interval; P =Probability value; LCA =Left color area; LTA =Left total area; RCA= Right color area; RTA = Right total area; TCA = Total color area; TFA = Total feather area; LCA:LTA= ratio of left color area to left total area; RCA:RTA= ratio of right color area to right total area; TCA:TFA = ratio of total color area to total feather.

Table S8: T-test results of dorsal feather length traits

	Male, n=142		Female, n=178			**95% CI**		**P**
**Parameters**	M (cm)	SD	M (cm)	SD	**t**	**Lower**	**Upper**	
LCL	3.87	0.68	3.219	0.49	8.92	0.45	0.71	0.000
RCL	3.79	0.67	3.35	0.48	9.67	0.49	0.75	0.000

FL	5.06	0.33	4.50	0.37	13.91	0.48	0.63	0.000
FRL	0.13	0.15	0.18	0.17	-3.18	-0.09	-0.02	0.002
TFL	4.94	0.44	4.89	0.45	11.96	0.42	0.58	0.000
**ratios**								
LCL :FL	0.76	0.12	0.73	0.09	2.78	0.02	0.06	0.006
RCL :FL	0.79	0.12	0.74	0.09	3.39	0.02	0.09	0.001
LCL : TFL	0.75	0.12	0.73	0.43	0.35	-0.06	0.09	0.724
RCL: TFL	0.77	0.12	0.74	0.40	0.68	-0.04	0.09	0.498
FRL:TFL	0.023	0.02	0.032	0.04	-4.77	-0.02	-0.01	0.000
FL: TFL	0.98	0.02	0.96	0.04	4.77	0.01	0.02	0.000

M =Mean; SD =Standard Deviation; t = t-test value; CI = Confidence Interval**;** P =Probability value; LCL= left color length; RCL=Right color length; FL= Feather length; FRL= Feather root length; TFL= Total feather length; LCL:FL= ratio of left color length to feather length; RCL:FL= ratio of right color length to feather length; LCL:TFL = ratio of left color length to total feather length; RCL:TFL= ratio of right color length to total feather length; FRL:TFL = ratio of feather root length to total feather length; FL:TFL= ratio of feather length to total feather length.

Table S9: Results of GO enrichment analysis for identified candidate genes

GO term	ID	P-value	FDR P-value1	Gene
Integral component of membrane	GO:0016021	3.12×10^{-7}	5.30×10^{-4}	VAPA, SEC63,
Cytoplasm	GO:0005737	4.32×10^{-6}	3.40×10^{-3}	PSTPIP1, SESN1, STK3,
Substrate adhesion-dependent cell spreading	GO:0034446	1.04×10^{-5}	3.40×10^{-3}	PEAK1, RAB1A, TMEFF2, ITGAV, NRP1
Positive regulation of GTPase activity	GO:0043547	1.09×10^{-5}	3.40×10^{-3}	DENND1A, DOCK1, RGS4, RTN4R, ZC3H15, RGS1, DENND1B, NRP1
Nucleus	GO:0005634	1.18×10^{-5}	3.40×10^{-3}	STK3, RALYL, ZNF704, TENM4, MEIS1, PURG, PTPRE, AGL, RGS4, ATF6, ONECUT1, POU2F1, CCNI, PANK4, ZC3H15, WRN, PKP2, BABAM2, ATF3, RXRG, PPP2CB, CDKL2, USP24, DOK1, KLF12, CAMTA1, CDK12, LHX9, DDX11, MAGI2
ATP binding	GO:0005524	1.20×10^{-5}	3.40×10^{-3}	PEAK1, STK3, PRKG1, ASNS, NEK7, PANK4,

GO term	ID	P-value	FDR P-value1	Gene
				WRN, TTF2, HSPA13, TADA1, CDKL2, HNRNPU, RAD51B, CDK12, DDX11, GUCY2F
Cell migration	GO:0016477	1.64×10^{-4}	1.46×10^{-2}	DOCK1, RAB1A, TMEFF2
Neuronal cell body	GO:0043025	1.80×10^{-5}	4.08×10^{-3}	DENND1A, RTN4R, GDPD5
Cellular response to hormone stimulus	GO:0032870	1.07×10^{-3}	4.70×10^{-2}	ROBO2, SLIT3
Plasma membrane	GO:0005886	5.04×10^{-5}	9.52×10^{-3}	PSTPIP1, VAPA
G protein-coupled glutamate receptor signaling pathway	GO:0007216	1.01×10^{-4}	1.19×10^{-2}	DENND1A
Dendritic spine	GO:0043197	1.43×10^{-4}	1.43×10^{-2}	PEAK1
Integral component of plasma membrane	GO:0005887	2.09×10^{-4}	1.74×10^{-2}	PEAK1
Neuromuscular process controlling balance	GO:0050885	2.15×10^{-4}	1.74×10^{-2}	TSPAN3
Signal transduction	GO:0007165	3.48×10^{-4}	2.29×10^{-2}	PSTPIP1
Regulation of Rho protein signal transduction	GO:0035023	3.49×10^{-4}	2.29×10^{-2}	STK3
Cytosol	GO:0005829	3.54×10^{-4}	2.29×10^{-2}	DENND1A
Phosphatidylinositol phosphate binding	GO:1901981	8.50×10^{-4}	4.13×10^{-2}	DENND1A
Protein phosphorylation	GO:0006468	9.44×10^{-4}	4.33×10^{-2}	PEAK1, STK3, NEK7, GUCY2F, CDK12, CDKL2, PRKG1
Protein serine/threonine phosphatase activity	GO:0004722	6.90×10^{-5}	1.07×10^{-2}	LRRC39, PPP2CB, CDC14A
Calcium ion binding	GO:0005509	8.72×10^{-5}	1.18×10^{-2}	PCDH9, SCUBE3, LRP8, MASP1, NOTCH2, TBC1D9B, SLIT3, UNC13C, SPOCK1, CAPN9

GO term	ID	P-value	FDR P-value1	Gene
Focal adhesion	GO:0005925	1.28×10^{-4}	1.362×10^{-2}	ITGAV, TADA1, NRP1
Neuron projection	GO:0043005	1.03×10^{-3}	4.55×10^{-2}	STK3
Nucleoplasm	GO:0005654	1.27×10^{-3}	4.81×10^{-1}	SESN1, DENND1A
Protein serine/threonine kinase activity	GO:0004693	3.60×10^{-2}	2.53×10^{-1}	CDK12, CDKL2

FDR, false discovery rate, using Benjamini and Hochberg method.

Table S10: Results of KEGG enrichment pathway for identified associated genes

Gene	Pathway
VAV3	Focal adhesion
ALDH1A3	Metabolic pathways, tyrosine metabolism pathway, Phenylalanine metabolism
ASNS	Biosynthesis of amino acids pathway, Metabolic pathways, Alanine, aspartate and glutamate metabolism
INPP5A	inositol phosphate metabolism, Phosphatidylinositol signaling system, Metabolic pathways
WNT3A	melanogenesis pathway
AGL	Metabolic pathways
B4GALNT3	Metabolic pathways
UROS	Metabolic pathways
MDH2	Metabolic pathways, Cysteine and methionine metabolism
PANK4	Metabolic pathways
CSGALNACT1	Metabolic pathways
ACACA	Metabolic pathways

Gene	Pathway
GUCY2F	Metabolic pathways
DBT	Metabolic pathways

Table S11: SNPs with a pairwise $r^2 > 0.4$ with the leader SNP at chr11: 19791413 bp

CHR_B	BP_B	SNP_B	r^2
11	19791413	snp13014847	1
11	19796049	snp13014935	0.553646
11	19843385	snp13015595	0.518605
11	19831322	snp13015478	0.517133
11	19790324	snp13014817	0.498257
11	19790325	snp13014818	0.498257
11	19787442	snp13014732	0.494478
11	19801194	snp13015004	0.477453
11	19812348	snp13015224	0.458217
11	19787490	snp13014733	0.457801
11	19822622	snp13015386	0.455626
11	19787109	snp13014716	0.450876
11	19787109	snp13014716	0.450876
11	19841207	snp13015565	0.444914
11	19843073	snp13015589	0.432326
11	19877499	snp13016373	0.430448
11	19797796	snp13014960	0.430025
11	19805443	snp13015113	0.429052
11	19786827	snp13014710	0.428403
11	19797789	snp13014959	0.420744
11	19805435	snp13015112	0.41801
11	19831289	snp13015477	0.415967
11	19805418	snp13015111	0.40756
11	19852185	snp13015805	0.405207
11	19793298	snp13014870	0.403433
11	19860513	snp13015991	0.402408

Supplementary Figure

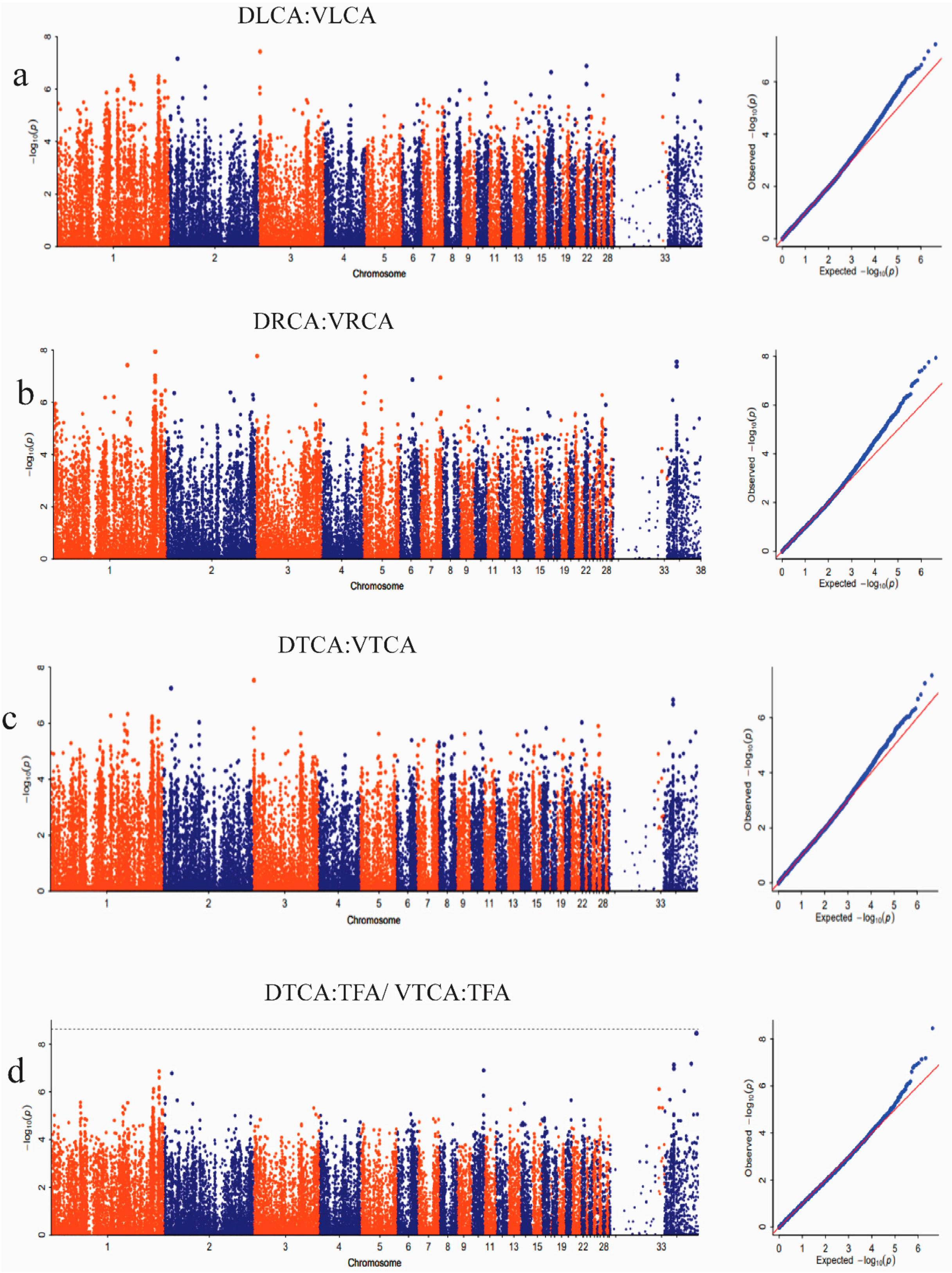
DLCA:VLCA
a
Chromosome
Expected $-\log_{10}(p)$
Observed $-\log_{10}(p)$
DRCA:VRCA
b
Chromosome
DTCA:VTCA
c
Chromosome
DTCA:TFA/ VTCA:TFA
d
Chromosome

Figure S1: Manhattan and QQ plots for countershading parameters. (a) DLCA: VLCA. (b) DRCA: VRCA. (c) DTCA: VTCA. (d) DTCA: TFA/ VTCA: TFA). The *x*-axis depicts the physical locations of each SNP along the chromosome, while the *y*-axis depicts the $-\text{Log}_{10}(p)$ values of SNP. The gray dotted lines signify the Bonferroni threshold level (correction threshold = 8.59).
